# User Engagement with A Multimodal Conversational Agent for Self-Care and Chronic Disease Management: A Retrospective Analysis

**DOI:** 10.1007/s10916-025-02202-2

**Published:** 2025-06-09

**Authors:** Selahattin Colakoglu, Mustafa Durmus, Zeynep Pelin Polat, Asli Yildiz, Emre Sezgin

**Affiliations:** 1 Department of Clinical Development, Albert Health, Istanbul, Turkey; 2 School of Medicine, Biruni University, Istanbul, Turkey; 3 The Abigail Wexner Research Institute, Nationwide Children’s Hospital, Columbus, OH USA; 4 The Ohio State University College of Medicine, Columbus, OH USA

**Keywords:** Conversational Agent, Chronic Disease Management, Voice Assistant, mHealth

## Abstract

**Introduction:**

Understanding user engagement with conversational agents is key to their sustainable use in mobile health and improving patient outcomes. This retrospective study analyzed interactions with a multimodal conversational agent in the Albert Health app to identify usage patterns and barriers to long-term engagement in self-care and chronic disease management.

**Methods:**

We retrospectively analyzed interactions from 24,537 users of a Turkish-language mobile health app (between January 1, 2022, and December 31, 2023). Interactions with the app’s multimodal conversational agent (voice and text) were categorized by demographics, interaction type, and engagement mode. Descriptive statistics summarized patterns, while Mann-Whitney U, Chi-square, and logistic regression identified group differences and predictors of sustainable engagement.

**Results:**

Most users were female (56%) and aged 30–45 (44%). The majority (92%) used general health programs, with only 8% in disease-specific ones. Common interaction types included health information (32%), small talk (20%), and clinical parameter logging (16%; e.g., blood pressure). Voice use was frequent in fallback (80%; unclear/ out-of-scope input), small talk (64%), and medication tasks (53%), while screen input was more common for clinical logging (61%) and health queries (59%). Engagement peaked in the first week and declined after 10 days. Sustainable engagement was associated with disease-specific program use (OR = 0.67, 95%CI: 0.60–0.74, *p* < 0.001), greater voice interaction (OR = 1.005, 95%CI: 1.004–1.006, *p* < 0.001), and a balanced mix of clinical and non-clinical use (OR = 1.56, 95%CI: 1.43–1.70, *p* < 0.05).

**Conclusions:**

This study highlights user preferences for voice interaction and health information access when using a multimodal conversational agent. The high rate of single-session users (58%) points to barriers to sustainable engagement, emphasizing the need for better user experience strategies.

## Introduction

The increasing demands on healthcare systems, the rising prevalence of chronic diseases, and the shift toward personalized patient care call for innovative solutions [1]. Conversational agents (CAs) offer a promising approach by automating routine interactions and enhancing healthcare delivery [2]. They have been shown to support patient self-management, assist clinical decision-making, and facilitate disease monitoring through data collection, personalized feedback, and easy access to health information [3].

With advances in eHealth and mobile health (mHealth) applications powered by artificial intelligence (AI), CAs have improved health communication and patient engagement [4]. They have been explored across a range of clinical applications, including screening, monitoring, patient education, and lifestyle coaching [5–8]. CAs also show promise for integration into clinical practice by working alongside healthcare professionals to monitor and support patients, streamline workflows, and promote health—ultimately aiming to reduce costs, improve efficiency, and enhance care quality [9]. CAs are increasingly used in telemedicine to perform routine check-ins and support healthcare tasks, especially in chronic disease management. These functions include symptom tracking and medication adherence when embedded into clinical workflows [9]. Additionally, CAs contribute to patient-generated health data and shared decision-making by informing healthcare providers about health events that occur outside clinical settings [10, 11]. Further evidence supports the use of CAs in self-care and chronic disease management (CDM). Their benefits include enhancing patient and caregiver engagement with health information [12], communicating medical test results [13], supporting post-intervention follow-ups [14] addressing health-seeking behaviors to improve outcomes [2, 15, 16], educating patients and caregivers, and delivering personalized health information [2, 17]. Studies also report that users perceive CAs as non-judgmental and accessible, which may improve access to health information via digital platforms [18]. Despite these benefits, most existing literature on CA use in CDM and self-care consists of small-scale feasibility studies (e.g., pre–post or quasi-experimental designs) and is primarily conducted in English [19, 20]. Additionally, previous research has often relied heavily on self-report measures, and a number of studies lack longitudinal data on real-world usage, focusing instead on short-term adoption rather than sustainable engagement [12, 19, 21, 22]. This limits the generalizability and applicability of findings to broader, more diverse populations and hinders the understanding of long-term user engagement.

To address this gap, we conducted a retrospective study examining usage patterns of a multimodal (voice and text) CA embedded in a mobile application (Albert Health app) designed for general health and chronic disease management [23]. This study aims to generate insights into the value and usage of CAs in healthcare and identify patterns that promote user engagement and retention. Our research questions are: (1) What are the characteristics of user interactions with a CA for self-care and chronic disease management? and (2) What are the engagement patterns and how do they relate to characteristics of CA usage over time? Our goal is to contribute to the growing body of evidence on CA adoption in healthcare and offer practical insights to improve patient engagement.

## Methods

This study analyzed a two-year retrospective dataset of de-identified user interactions with the Albert Health app, collected from both iOS and Android platforms in Turkish.

### Recruitment and Study Setting

We included users who engaged with the conversational agent (referred to as “Albert” hereafter) through the Albert Health app on either iOS or Android platforms between January 1, 2022, and December 31, 2023. The app supports the Turkish language and is primarily used in Turkey.

Users accessed the app through three primary channels: (1) downloading it independently from app stores, (2) receiving invitations from healthcare professionals during hospital visits, and (3) being referred by insurance companies as part of health management programs. Inclusion criteria required users to be located in Turkey and to have interacted with the conversational agent at least once after completing the initial tutorial. Users who entered an invalid birthdate (e.g., before 1900 or after their registration date) were excluded from the analysis.

During installation, users were prompted to provide informed consent for the use of their data for research purposes. Upon consenting, they completed an onboarding survey that collected demographic information and details about any health conditions. To encourage continued engagement, a one-time reminder notification was sent on the eighth day after installation.

### Mobile Health Assistant

**Fig. 1:**
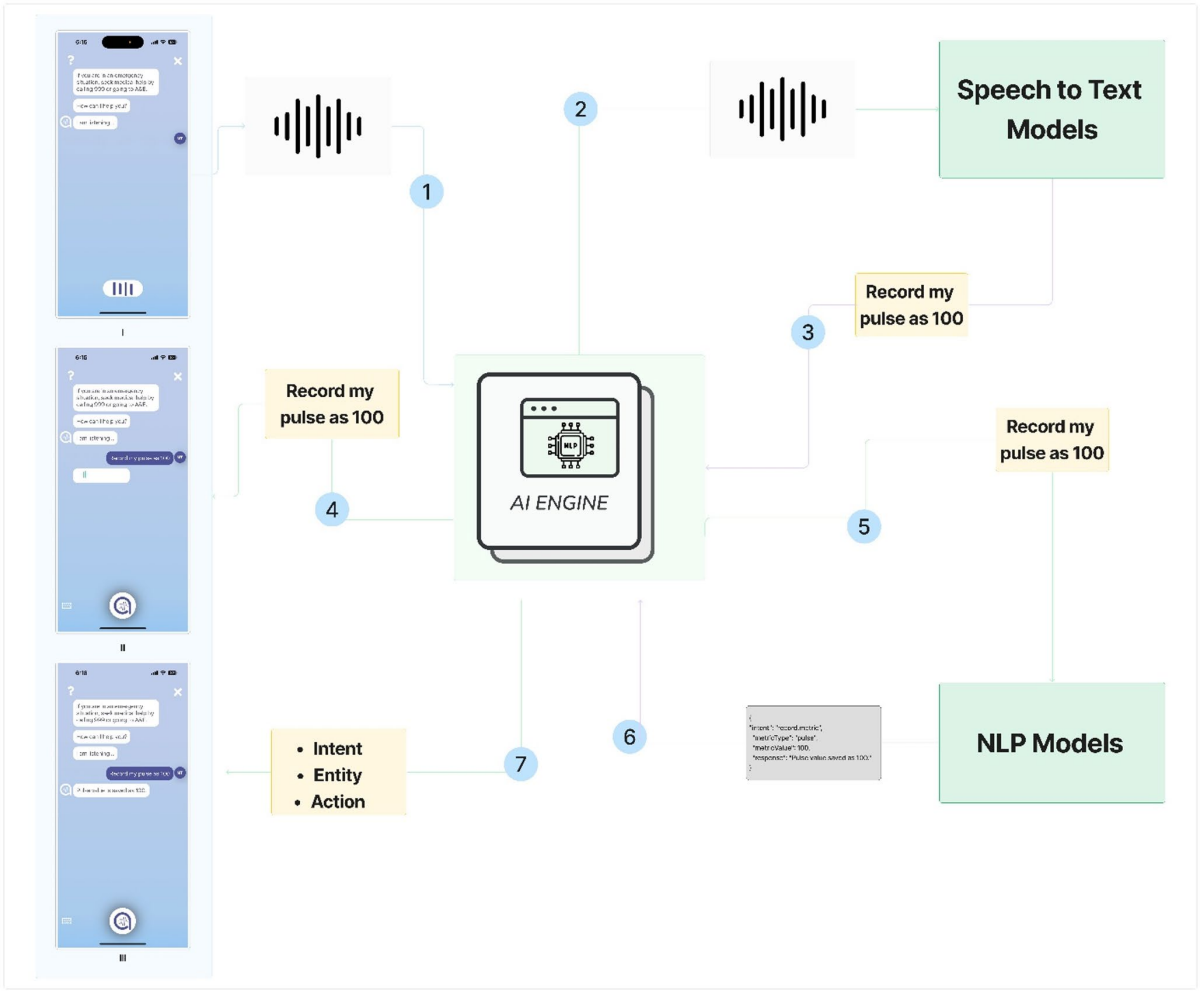
User engagement flow

Albert is a digital health platform developed by Albert Health. It comprises multiple health and chronic disease management programs and is freely available for download from the Google Play Store and Apple App Store. As of 2023, Albert has served approximately 150,000 users through partnerships with public and private healthcare institutions and industry stakeholders [24]. The app functions as a multimodal health assistant, offering both voice and screen-based interaction. Screen-based interactions include text input and the use of assistive buttons (initiated by user or in response to app notifications). Both interaction modes (voice and screen-based) required the same number of steps to complete tasks within the CA interface. Albert is built on conversational development platforms, including Google Dialogflow and Rasa, [25, 26] which provide natural language processing (NLP) capabilities such as intent recognition (i.e., identifying the user’s goal or request) and entity extraction (i.e., detecting specific information such as medication names or symptom details). To enable voice input and output, the app integrates Google Speech-to-Text (STT) and Google Text-to-Speech (TTS) services. Voice responses are delivered in a male voice and enhanced using Speech Synthesis Markup Language (SSML) to produce natural and expressive output. These components are orchestrated by the app’s central processing system, referred to as the AI Engine, which integrates NLP models (Rasa and machine learning frameworks provided by Dialogflow) and STT services. The AI Engine receives user audio in streaming format, converts it to text via STT, and then passes the transcribed text to the NLP models for interpretation and guidance. Figure 1; Table 1 illustrate the flow of user engagement and provide a sample interaction to explain this process.

In addition to the CA, Albert provides alternative methods for accessing health information. Users can browse pre-organized content through traditional navigation tabs (such as a library module with a limited number of health-related articles). While these navigation paths offer structured access to information, they require users to know what they are looking for and navigate through multiple screens. In contrast, the CA allows users to directly request specific information through natural language. Within the scope of this study, we did not report engagement with non-conversational features of Albert.

**Table 1:** User interaction example in line with Fig. 1

1. The user initiates an interaction with the mobile application using a voice command, such as “Record my pulse as 100.” The app interface captures this audio input.
2. The audio is transmitted to the Google Cloud Speech API, which uses advanced machine learning models to transcribe spoken language into text.
3. The transcribed text—“Record my pulse as 100”—is returned from the Google Cloud Speech API to the application’s processing system.
4. The application displays the transcribed text on the user interface for visual confirmation.
5. The text is then sent to the AI Engine, which forwards it to the natural language processing (NLP) model for structural and semantic analysis.
6. The NLP model identifies the user’s intent (e.g., to record health data), extracts relevant entities (e.g., the pulse value), and determines the appropriate action (e.g., logging the pulse in the user’s health record).
7. These components—intent, entities, and actions—are processed by conversational AI frameworks such as Rasa or Dialogflow. These frameworks manage the dialogue, maintain context, and ensure accurate execution of the user’s command.
8. Once the intent and entities are validated, the app records the user’s pulse rate as 100 in their personal health record. The interface then provides visual feedback to confirm that the command was understood and successfully completed.

Albert offers multiple programs tailored to either general health management (GEN) or disease-specific self-management (DMP). Each program includes its own version of the CA, adapted from the core Albert system to address specific user needs. DMPs focus on managing particular health conditions—such as diabetes, hypertension, heart failure, and asthma—by providing condition-specific educational content and enabling users to record relevant clinical parameters. In contrast, GEN programs provide general health support and lifestyle guidance without targeting a specific disease.

### Intents

We categorized each user–CA conversation into six intent groups based on whether the interaction was clinical (Health Information, Medication-related, Clinical Parameter) or non-clinical in nature (Fallback, Small Talk, and Technical Queries/Support). Interactions involving questions about health topics or access to care—such as FAQs, wellness tips, pharmacy locations, appointment scheduling, or general advice—were grouped under Health Information. Conversations related to medication usage, schedules, or recording were classified as Medication-related. Instances in which users logged clinical data (e.g., blood pressure, blood glucose, body mass index, or symptom journals) were assigned to the Clinical Parameter group. The Fallback category captured responses triggered when the CA was unable to understand the user’s input. Small Talk included casual or social interactions, and Technical Queries/Support encompassed questions related to app use or troubleshooting. (Table 2).

**Table 2:** Intent groups, explanations and examples

Intent Category	Definition	Conversational examples	
		GEN (General health management)	DMP (disease-specific self-management)
Health Information	Interactions about general health advice, or medical knowledge that could be relevant to both general well-being and specific health conditions. Example: - Ask health-related questions - Show pharmacy on duty - Get a telehealth appointment	User: What’s an ideal body fat percentage for women? CA: Ideal body fat percentages vary by individual and depend on factors like age and fitness level. Generally, for women, a body fat percentage of 21–24% is considered healthy. Athletes might have lower percentages, around 14–20%, while levels above 32% might be considered higher than optimal for health. It’s important to focus on overall well-being and consult with a healthcare professional for personalized advice.	User: I’m not sure if my inhaler technique is right. CA: Ensuring the correct technique is crucial for your treatment. Could you tell me the name of your inhaler? User: Ventolin HFA. CA: I’ll send you a video for Ventolin HFA inhalers. If you have any questions after watching, I’m here to help!
Clinical Parameters	The process of tracking, recording, and reviewing health metrics such as blood pressure, blood glucose level, or other clinical data Example: -Record blood pressure or glucose level - Measure BMI -Record a symptom diary	User: Can you calculate my BMI? CA: Absolutely. Please provide me with your height and weight. User: I’m 175 cm and weigh 70 kilos. CA: Based on that, your BMI is 22.9, which is within the healthy weight range. If you want to schedule an online meeting with a dietitian, please let me know.	User: I want to log my blood pressure. CA: Please tell me your latest reading. User: It’s 13 over 8. CA: I’ve recorded your blood pressure as 130/80 mmHg.
Medication- related	Actions related to the management and understanding of medications, supplements, or treatments, applicable to routine health maintenance or condition-specific management. Example: - Record medication - Mark medication as taken - Show daily medications	User: Can you show my daily medication schedule? CA: Certainly. − 08:00: Vitamin D, 1000 IU with breakfast. − 18:00: Omega-3 supplement with dinner.	User: I need to record my new medication. CA: Of course. Please tell me the name of the medication. User: Metformin. CA: Got it. I’ve added Metformin to your medication list. What time will you be taking it?
Fallback	Responses when CA is unable to understand the intent of user	User: [Attempts to speak, but the speech is unclear and not transcribed correctly] CA: I’m sorry, I didn’t catch that. Could you please repeat what you just said? User: [Attempts to speak again, but the speech is still unclear and not transcribed correctly] CA: It seems we’re having trouble understanding your message. I apologize for the inconvenience. If you’re experiencing an urgent issue, it’s important to contact a healthcare professional directly or dial emergency services. Remember, this platform is not a substitute for professional medical advice, diagnosis, or treatment. Please try to rephrase your concern or reach out to your healthcare provider for immediate assistance.	
Smalltalk	Casual interactions and system guidance and navigation	User: How are you? CA: I’m always here and ready to help! Thanks for asking. How are you feeling today? User: I am excited. CA: That’s great to hear! Is there something special happening today, or are you excited to try something new?	
Technical Support & Guidance	Questions related to app use, issues or troubleshooting	User: The app keeps freezing. CA: I’m sorry for the inconvenience. Try restarting your device or reinstalling the app. Would you like step-by-step assistance, or would you prefer direct access to customer support? User: Customer support, please. CA: Absolutely! [Directs user to support section]	

### Data Privacy

To protect user privacy, all data were deidentified, and each user was assigned a randomly generated identifier at the time of registration. Encryption protocols were applied to secure the data throughout collection and storage. Given the retrospective nature of this study, the analysis was limited to the existing dataset, and no additional data were collected.

### Data Analysis

We analyzed user interactions data (i.e., each query) from users who completed the CA tutorial and used the chat function. Interaction data from tutorial sessions—which occur when a user downloads and initiates the app for the first time—were excluded. The dataset included user demographics, program participation, and detailed engagement metrics (e.g., number and types of interactions; mode of interaction—voice or screen-based interaction).

We first conducted descriptive statistics to summarize user demographics and usage patterns. The Shapiro-Wilk test was used to assess normality. As the data were not normally distributed, we applied the Mann-Whitney U test for group comparisons and calculated Cliff’s Delta to estimate effect size.

To examine whether interaction mode (voice vs. screen-based interaction) varied by gender, intent group, age group, or program group, we constructed contingency tables and used Pearson’s Chi-square tests, with Cramer’s V reported as the effect size.

Lastly, we conducted logistic regression to identify predictors of long-term engagement. Predictor variables included program group (DMP vs. GEN), frequency of speech versus screen-based interaction, internally calculated mean confidence score, age group, and level of clinical context in the interaction. The mean confidence score represents the CA’s natural language understanding confidence level for each user interaction, as provided by the underlying NLP platforms (Dialogflow and Rasa). This score ranges from 0 to 1, with higher values indicating greater confidence in correctly interpreting the user’s intent. The level of clinical context was categorized based on the ratio of clinical to non-clinical interactions: high (ratio > 1, indicating more clinical than non-clinical intents), medium (ratio = 1, indicating equal distribution), and low (ratio < 1, indicating fewer clinical than non-clinical intents). Clinical intents include Health Information, Medication-related, and Clinical Parameter categories as defined previously. Continuous variables were standardized, and Wald-based 95% confidence intervals (CIs) were derived for each odds ratio (OR). We assessed multicollinearity using variance inflation factors (VIFs), and model discrimination was evaluated by plotting the receiver operating characteristic (ROC) curve and calculating the area under the curve (AUC).

## Results

Between January 1, 2022, and December 31, 2023, a total of 24,537 users completed the Albert tutorial and generated 113,780 interactions with the CA.

### Demographics

Of the 24,537 users, 56% of those who reported their gender (*n* = 15,723) identified as female, and 44% identified as male. Additionally, 36% of the total sample (*n* = 8,814) did not report their gender. Regarding age, 31% did not disclose their age. Among those who did, 6% (*n* = 1,012) were aged 0–15, 30% (*n* = 4,673) were 15–30, 44% (*n* = 7,584) were 30–45, 17% (*n* = 3,035) were 45–60, and 3% (*n* = 568) were 60 or older (see Table 3).

The Shapiro-Wilk test (*p* < 0.001) indicated non-normal distribution for age, gender, and program type. A binomial test was conducted to assess whether the gender distribution deviated significantly from a 50/50 split. Results showed that female users (56%) were significantly more likely to engage with the CA than male users (*p* < 0.0001).

**Table 3:** User demographics & program type

Demographics	Number of Users	Percentage
**Gender (** ***n*** ** = 15,723)**		
Female	8,805	56%
Male	6,918	44%
**Age (** ***n*** ** = 16, 872)**		
0-15^1^	1,012	6%
15–30	4,673	28%
30–45	7,584	45%
45–60	3,035	18%
60 or more	568	3%
**Program Type (** ***n*** ** = 24,537)**		
GEN users- non-specific to any health condition	22,629	92%
DMP users- with a specified health condition	1,908	8%

^1^ Users who indicated an age between 0–15 years are presumed to represent accounts created by parents or caregivers on behalf of children

### Health Condition-Specific Findings

Among all participants, 7.75% (*n* = 1,901) were enrolled in a DMP, accounting for 8.4% of total interactions and averaging 5.03 interactions per user. In contrast, 92.2% (*n* = 22,628) participated in a GEN program, contributing 91.6% of total interactions, with an average of 4.60 interactions per user (Table 4).

A Mann-Whitney U test (W = 20,739,146, *p* = 0.008) indicated a statistically significant difference in interaction frequency between DMP and GEN groups. However, the effect size was negligible (Cliff’s Delta = − 0.0357, 95% CI: − 0.0634 to − 0.0080), suggesting that although DMP users engaged slightly more frequently, the practical difference was minimal.

**Table 4:** Engagement rates and program

Program Type	Mean Interaction per User	Mean Session Count per User
General Health Management (GEN)	4.60	2.07
Disease management program (DMP)*	5.04	2.89
**Gender (** ***n*** ** = 15,723)**		
Female	5.14	2.27
Male	4.85	2.38
**Age by years (** ***n*** ** = 16, 872)**		
0-15^1^	5.30	2.07
15–30	4.27	1.94
30–45	4.77	2.12
45–60	5.84	2.74
60 or more	6.97	4.06

*Disease management programs include following therapeutic areas: Cardiometabolic, Respiratory, Neurology, Nutrition, Oncology, Rare Diseases and Women’s Health

### Engagement and Interaction

Users interacted with Albert most frequently to access health information (32%), followed by logging clinical parameters (16%) and addressing medication-related topics (12%) within the clinical context (Table 5).

**Table 5:** Interaction categories and initiation methods

Category	Number of Interactions (Frequency%)	Initiated by Screen - (text, notification, assistive button)	Initiated by Voice - (speech)
Health Information	36,910 (32%)	59%	41%
Clinical Parameters	18,350 (16%)	61%	39%
Medication-related	13,416 (12%)	47%	53%
Technical Support	8,284 (7%)	50%	50%
Smalltalk	22,246 (20%)	36%	64%
Fallback	14,574 (13%)	20%	80%
*Total*	113,780	48%	52%
**Gender (76**,**962)**			
Female	45,298 (59%)	47%	53%
Male	31,664 (41%)	53%	47%
**Age Group (82**,**377)**			
0–15	5,345 (7%)	42%	58%
15–30	19,982 (24%)	48%	52%
30–45	35,766 (43%)	49%	51%
45–60	17,347 (21%)	52%	48%
60+	3,937 (5%)	61%	39%
**Program Group**			
General Health Management (GEN)	104,155 (91%)	48%	52%
Disease management program (DMP)	9,625 (9%)	46%	54%

Regarding the mode of interaction, females used voice slightly more often (53.1%), whereas males showed a modest preference for screen-based interaction (52.6%) (χ²(1) = 248.89, *p* < 0.001). Marked differences appeared across intent types: fallback (80.2%) and small talk (64.5%) were predominantly voice-driven, while clinical parameter interactions were mostly initiated via screen-based interaction (60.9%) (χ²(15) = 11,681, *p* < 0.001). Similarly, non-clinical intents occurred more frequently via voice (66.9%), while clinical tasks were more often completed through screen-based interaction (χ²(3) = 6,457.68, *p* < 0.001).Age-related trends were also observed. Younger users (ages 0–15) favored voice input, whereas older users (60+) were more likely to use screen-based interaction, though the effect was modest (χ²(4) = 330.55, *p* < 0.001). Users enrolled in disease management programs showed a higher preference for voice (54.3%) compared to those in general health programs (52.3%) (χ²(1) = 14.24, *p* < 0.001). Intent type showed the strongest observed association with interaction mode (Cramér’s V = 0.28), consistent with a moderate effect size. Through a manual review of a random subsample (*n* = 200), we identified that the majority (approximately 80%) were due to non-meaningful speech or slang usage. In addition, 10% of these instances involved users terminating the session prematurely while the remaining 10% were attributable to the speech-to-text system failing to capture specific medical terminology or infrequently used drug names.

**Fig. 2:**
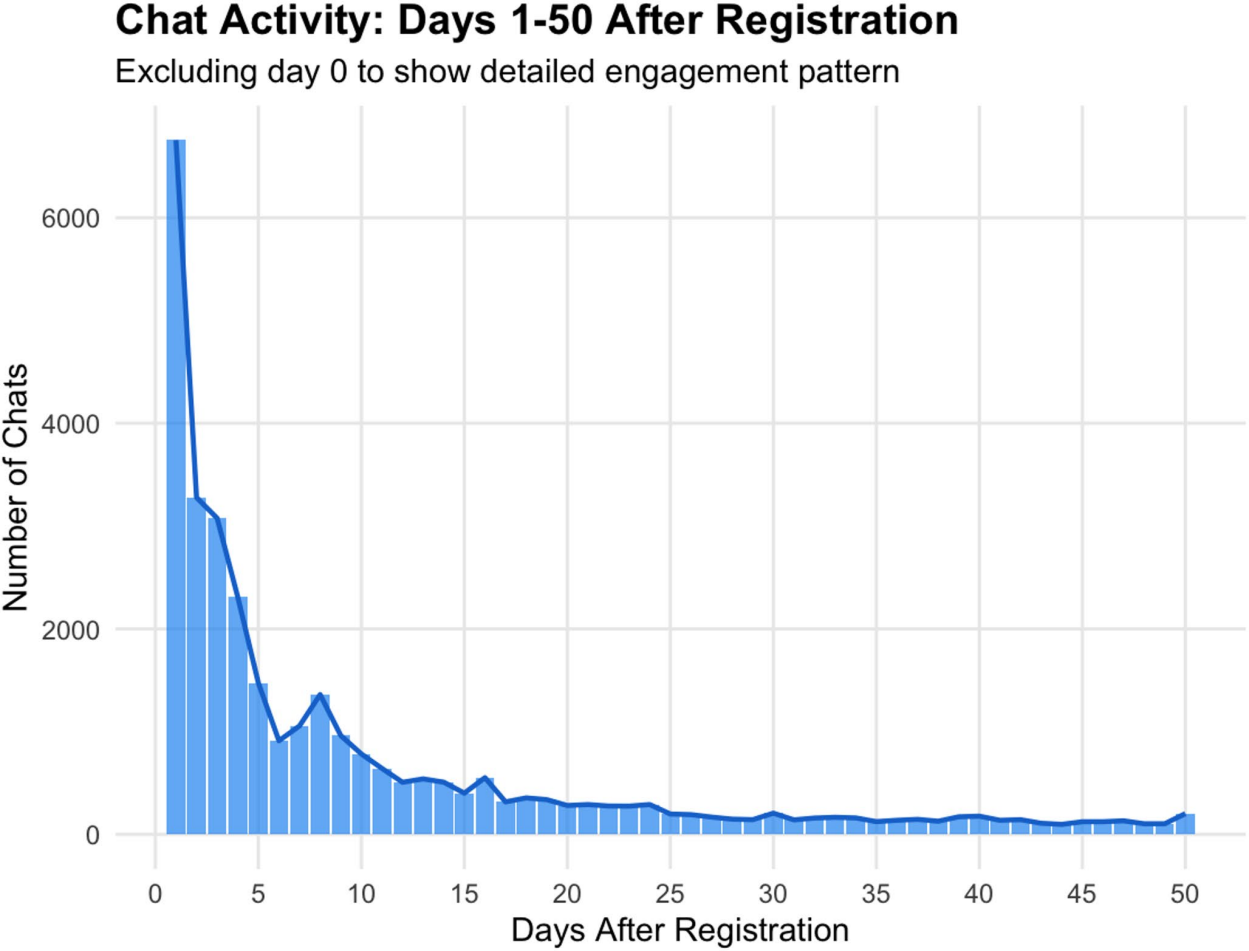
Trend in daily usage within first 50 days in number of chat sessions

User engagement frequency varied notably over time. Interactions peaked sharply on the first day after registration and declined rapidly within the first 10 days. Of the total user base, 14,194 users (58%) completed only one session, while 10,335 users (42%) engaged in two or more sessions (Fig. 2). A secondary peak occurred around day 8, corresponding with the scheduled reminder notification, followed by a stabilization at lower engagement levels. This distinction between single-session and multi-session users was used as a key outcome in the logistic regression analysis.

### Logistic Regression Analysis

The logistic regression model (see Table 6) demonstrated moderate discriminative performance (Residual deviance = 31,890, AIC = 31,914, AUC = 0.65). Gender did not have a meaningful effect on long-term usage and was therefore excluded from the final model. Membership in the GEN group was associated with lower odds of long-term engagement compared to the DMP group (OR = 0.67, 95% CI: 0.60–0.74, *p* < 0.001). A higher percentage of voice-based interactions was positively associated with long-term engagement (OR = 1.005, 95% CI: 1.004–1.006, *p* < 0.001), while a higher percentage of screen-based interaction was associated with lower odds (OR = 0.994, 95% CI: 0.993–0.995, *p* < 0.001).

Interestingly, a higher mean confidence score in the CA’s responses was significantly associated with lower odds of sustainable engagement (OR = 0.43, 95% CI: 0.33–0.56, *p* < 0.001). Younger adults (ages 15–30 and 30–45) were significantly less likely to engage over time (OR ≈ 0.76–0.77), whereas older users (45–60, 60+) showed weaker or nonsignificant differences compared to the reference group. Medium clinical context (OR = 1.56) and low clinical context (OR = 1.07) both significantly increased the odds of long-term engagement relative to high clinical context. Variance inflation factors (VIF) for all predictors showed minimal multicollinearity (< 2).

**Table 6:** Logistic regression results

Coefficients*	Estimate	Std. Error	z value	Odds Ratio (OR)	95% Confidence Interval	*p*-value
Intercept	1.18299	0.16126	7.336	3.26	(2.38, 4.48)	< 0.001
Program Group (GEN vs. DMP)	-0.40450	0.05188	-7.796	0.67	(0.60, 0.74)	< 0.001
Speech %^†^	0.51192	0.06047	8.465	1.005	(1.004, 1.006)	< 0.001
Text %^†^	-0.59396	0.05860	-10.135	0.994	(0.993, 0.995)	< 0.001
Mean Confidence	-0.84621	0.13793	-6.135	0.43^‡^	(0.33, 0.56)	< 0.001
Age Group (15–30 vs. 0–15)	-0.27451	0.07022	-3.909	0.76	(0.66, 0.87)	< 0.001
Age Group (30–45 vs. 0–15)	-0.25902	0.06871	-3.770	0.77	(0.67, 0.88)	< 0.001
Age Group (45–60 vs. 0–15)	-0.01851	0.07532	-0.246	0.98	(0.85, 1.14)	0.81
Age Group (60 + vs. 0–15)	-0.19478	0.11333	-1.719	0.82	(0.66, 1.03)	0.086
Clinical Context (low vs. high)	0.06887	0.03180	2.165	1.07	(1.01, 1.14)	0.030
Clinical Context (medium vs. high)	0.44394	0.04558	9.740	1.56	(1.43, 1.70)	< 0.001

* The intercept represents the baseline log-odds when all other variables are at zero or their reference levels. Program Group (GEN vs. DMP) compares general health vs. disease management programs. Speech % and Text % represent the proportions of speech- and text-based usage. Clinical Context (low vs. high, medium vs. high) compares different levels of clinical relevance. Reference categories for categorical variables are: Program Group (DMP), Age Group (0–15), and Clinical Context (high). † Speech % and Text % β-coefficients reflect a 100%‐point (pp) change; the reported ORs are rescaled to a 1 pp increase (i.e., OR = exp(β ÷ 100)). ‡ Mean Confidence ranges from 0 to 1; the reported OR (0.43) corresponds to the full 0–1 shift

## Discussion

Our study presents a retrospective analysis of user interactions and engagement with a CA in the healthcare setting, highlighting patterns of use among patient populations—particularly in the contexts of chronic disease management, self-care, and general health monitoring.

The analysis of user demographics revealed higher engagement among female users, a trend that may reflect gender differences in health-seeking behaviors and the proactive use of mobile technology for health management. [27]. These findings are consistent with those of Darcy et al [28], whose study of over 36,000 participants reported that nearly 58% were female, while more than 11% declined to disclose their gender—similar to the non-disclosure patterns observed in our sample. The lower rate of age disclosure may reflect privacy concerns or a perception that age is not relevant to the app experience [29, 30]. Nevertheless, the reported age distribution aligned with prior CA studies, where most users were young or middle-aged adults, [3, 28] suggesting a comparable user profile for CA adoption in chronic disease management.

Notably, a small subgroup of users reporting an age of 0–15 years—likely accounts created by caregivers on behalf of children—tended to favor voice interactions, highlighting the app’s indirect utility in pediatric health contexts. Additionally, while older adults (45+) showed comparable engagement rates, younger adults (15–45) were significantly less likely to sustain long-term interactions.

Users preferred general health management programs over disease-specific ones—possibly due to a greater focus on preventive health measures [3]. The lower uptake of disease-specific programs may be attributed to their narrower scope and more restrictive enrollment criteria [31]. However, our finding that disease-specific program users demonstrated more engagement over time likely reflects the fact that users with specific health conditions have more complex ongoing health information and management needs, which may motivate continued use of supportive tools like conversational agents. Interestingly, users engaged in a wide range of in-app interactions beyond core clinical categories such as health information, medication, and clinical parameters. These included small talk, technical support, and guidance, suggesting that the versatility of the CA in addressing diverse user needs may contribute to its appeal [32]. Indeed, logistic regression results indicated that users who engaged in a balanced mix of clinical and non-clinical interactions had significantly better long-term retention compared to those whose usage was dominated by clinical intents (i.e., > 50% clinical interactions).

Manual review of fallback instances revealed that the most common cause of unrecognized input was either the absence of meaningful speech or the use of slang. We also anticipate that some fallbacks were due to technical or user-related issues, such as prematurely terminated speech, limitations in the speech-to-text system, or difficulty recognizing specific medical terminology or less common drug names (e.g., “Hodgkin’s lymphoma”). Similar challenges have been noted in prior healthcare CA research, where breakdowns in conversation were linked to limitations in text processing and contextual interpretation [33–35]. These findings highlight the need for ongoing evaluation and improvement of CA systems in healthcare contexts. Additionally, large language models may offer potential for improving natural language understanding, particularly when standard approaches fail to capture medical terminology [36].

When interactions were successful, voice emerged as the dominant mode of engagement, suggesting users’ natural adaptation to voice interfaces and their appreciation for hands-free accessibility. Logistic regression analysis further demonstrated that a higher proportion of voice-initiated interactions significantly increased the odds of sustainable use, whereas a greater reliance on screen-based interaction was associated with reduced engagement. Notably, users often initiated conversations with greetings or social inquiries, treating the CA as a human-like partner [37]. This observed preference for voice-based interaction—particularly evident in casual and fallback categories—may inform future app design enhancements by reflecting user preferences for convenience and potentially addressing accessibility needs [1, 38].

A substantial number of users (*n* = 14,194) engaged with the CA for only a single session, indicating potential barriers to sustainable use, such as unmet expectations, usability issues, or limited perceived value [39–41]. As previously noted, interaction mode and content balance influenced long-term engagement. Importantly, logistic regression revealed that higher mean confidence scores in CA responses were linked to lower odds of continued engagement. While counterintuitive at first glance—as one might expect confident responses to build trust—this finding may have several explanations. First, sustained users may engage with more complex or ambiguous health queries over time that inherently generate lower confidence scores compared to less complex initial interactions. Second, high-confidence responses might appear overly rigid, impersonal, or lacking in nuance, potentially diminishing perceived authenticity in contexts where users expect conversational or empathetic communication [42, 43]. Finally, highly confident but incorrect or partially relevant responses could frustrate users more than responses that acknowledge uncertainty when appropriate.

In contrast, the 10,335 users who engaged in two or more sessions represent a more committed segment, potentially reflecting ongoing perceived value in the app’s features. This highlights an opportunity for targeted user experience enhancements and personalization strategies aimed at converting single-session users into returning users. Varying levels of engagement may also reflect differing user needs or satisfaction with the information and support provided [44].

The initial spike in engagement immediately after registration—followed by a smaller peak around day 10, coinciding with a reminder notification—is consistent with common digital engagement patterns [45]. This likely reflects a period of initial curiosity and exploration, followed by a drop-off as novelty diminishes. Identifying factors that promote sustainable use beyond this early window may be critical to improving long-term adoption and the therapeutic value of health-related CAs. Potential strategies include personalized health insights, timely reminders for health tracking, or the integration of gamification elements to support ongoing user interaction [46–48].

### Limitations

This study has several limitations. First, the data are observational and derived from user interactions within a single mobile application, which limits the generalizability of the findings to other CAs or health apps. Second, reliance on self-reported demographic information —combined with a substantial proportion of users choosing not to disclose their age or gender—may introduce bias and reduce the accuracy of demographic insights. Additionally, the high proportion of single-session users may reflect selection bias, as the dataset primarily represents individuals who opted to engage with the CA at least once. The tendency to use other UI features (e.g., navigating to the app library section for reading materials) may inform the CA use behavior, however, other features were not investigated within the scope of this study. The imbalance between general health management and disease-specific user groups may have further limited comparative analyses across subgroups. Cultural and linguistic factors unique to the target population may also impact the generalizability of results to other settings. Moreover, the study did not assess the role of graphical user interface elements in promoting sustainable engagement. The lack of qualitative data on user satisfaction and reasons for discontinuation limits a more nuanced understanding of user experience. Future research should incorporate qualitative interviews or surveys to explore user disengagement and guide targeted design improvements. Finally, the retrospective design precludes causal inference regarding the relationship between demographics and engagement patterns.

## Conclusions

This study demonstrates both the potential and challenges of conversational agents in healthcare settings. Key findings include higher engagement among female users, preference for voice interaction across multiple user segments, and greater sustainability among disease management program participants compared to general health users. The rapid decline in engagement after initial interaction represents a challenge, as influencing factors being thebalanced clinical and non-clinical interaction, appropriate confidence calibration in CA responses, and program specificity. These insights provide a foundation for designing more effective digital health interventions that leverage conversational interfaces to support patient self-management while maintaining engagement beyond initial curiosity. Future development should focus on enhancing personalization, optimizing interaction modalities, and creating value that encourages sustainable use across diverse user populations.

## Data Availability

No datasets were generated or analysed during the current study.
